# Neuroendocrine Small Bowel Tumor

**DOI:** 10.5334/jbsr.4038

**Published:** 2025-09-17

**Authors:** Labinot Alija, Adelard De Backer

**Affiliations:** 1 Vrije Universiteit Brussel (VUB), Universitair Ziekenhuis Brussel (UZ Brussel), Department of Radiology, Laarbeeklaan 101, 1090 Brussels, Belgium; 2 Vrije Universiteit Brussel (VUB), Universitair Ziekenhuis Brussel (UZ Brussel), Laarbeeklaan 101, 1090 Brussels, Belgium

**Keywords:** CT, neuroendocrine tumor, small bowel, intestinal obstruction

## Abstract

*Teaching point:* CT *enterography/enteroclysis* allows adequate evaluation of neuroendocrine small bowel tumor and extra-enteric abnormalities.

## Case History

A 66-year-old woman presented with a long history of atypical abdominal cramps, constipation alternating with diarrhea, and weight loss. Clinical examination showed mild abdominal distension. Four adenomatous polyps in the colon were resected endoscopically.

CT enteroclysis showed a stenotic ileal loop centrally located in the lower abdomen with marked contrast-enhancing wall thickening. In the adjacent mesentery, a nodular mass with perilesional soft tissue strands indicating desmoplastic reaction was noted. Proximal dilation of small bowel loops with diffuse moderate wall with signs of low-grade mechanical obstruction was noted (Figures 1a–d). Surgical resection and histopathologic analysis revealed a well-differentiated neuroendocrine tumor (NET) of the ileum and a tumoral implant in the adjacent mesentery.

**Figure 1a:**
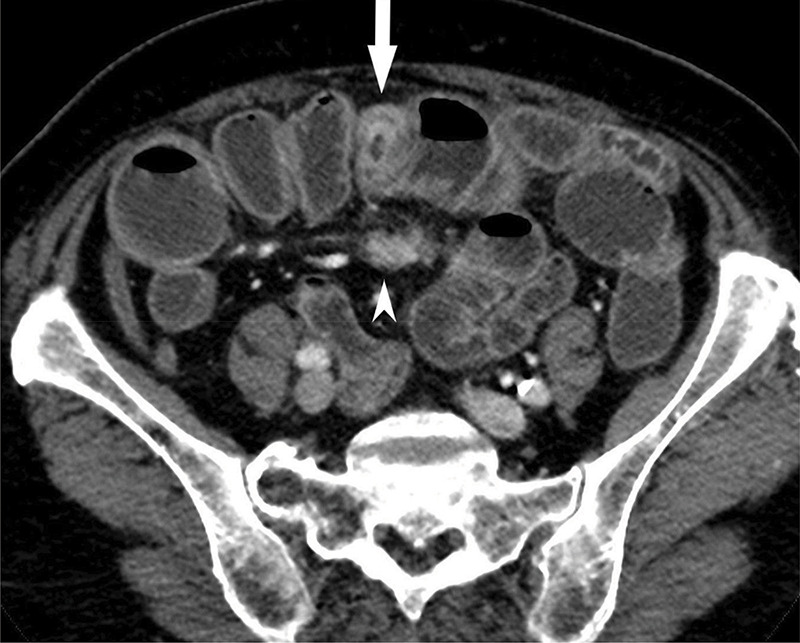
CT showing contrast-enhancing ileal NET (arrow) and mesenteric mass (arrowhead).

**Figure 1b:**
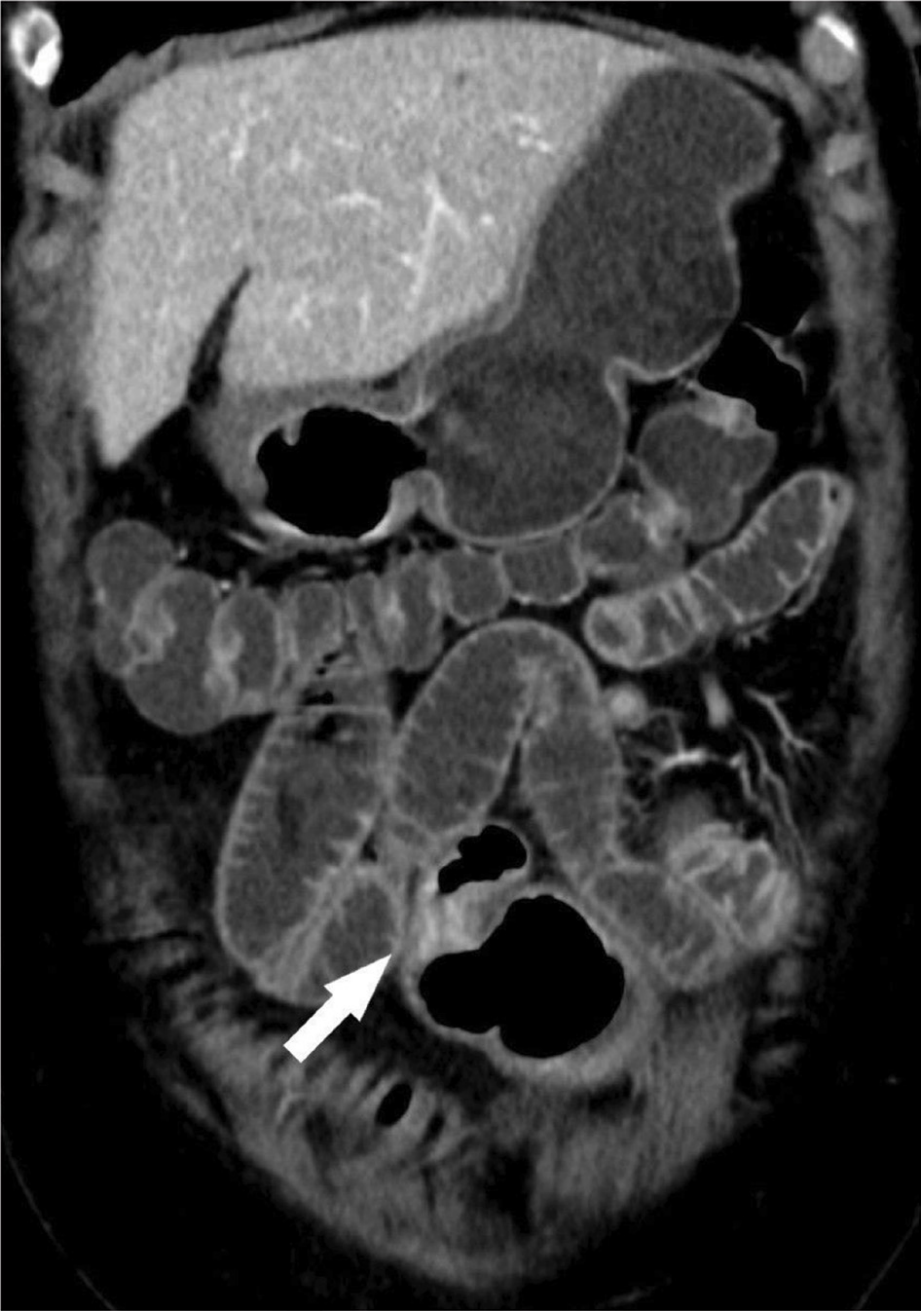
CT showing stenotic ileal loop with marked contrast-enhancing wall thickening.

**Figure 1c:**
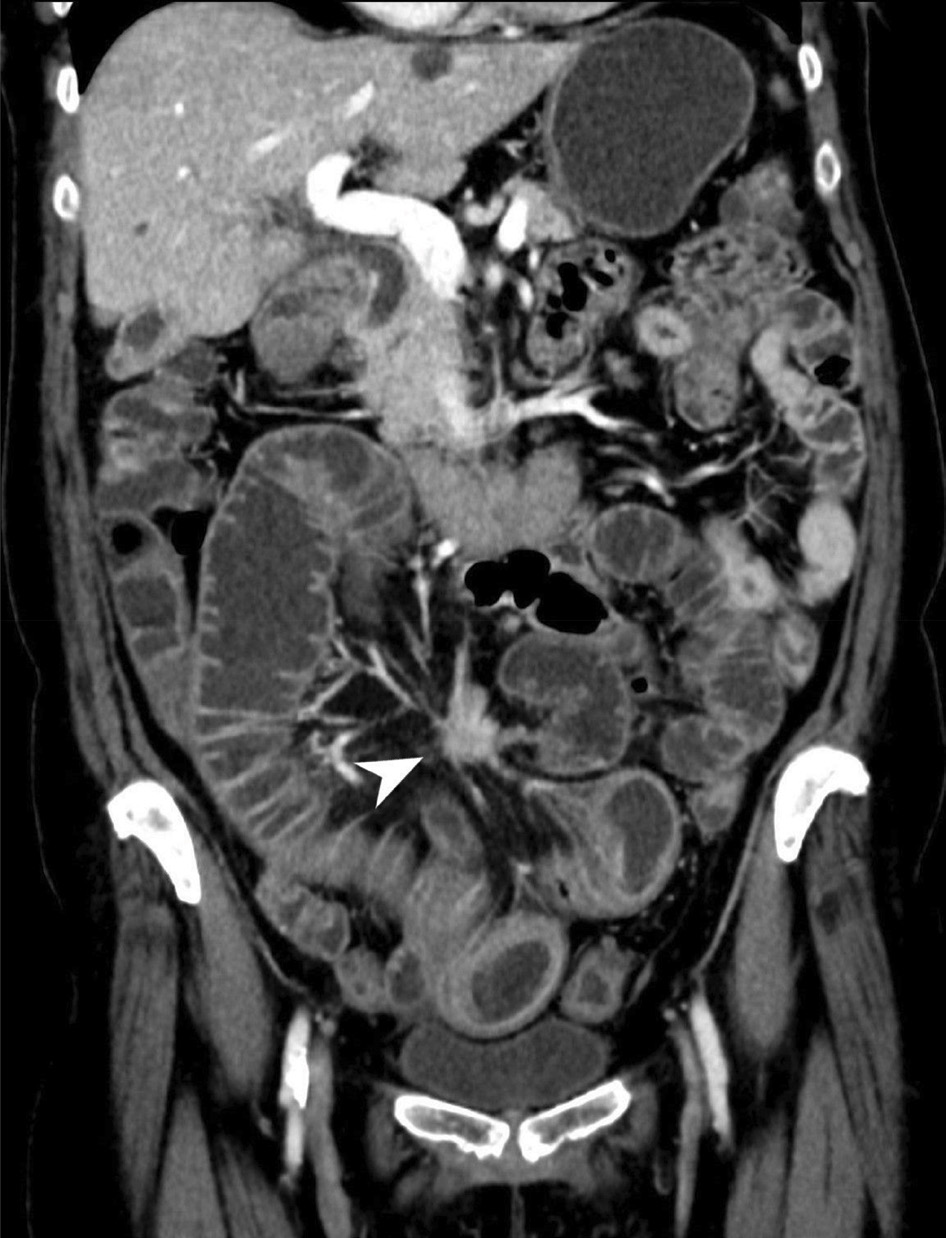
Mesenteric mass with perilesional soft tissue strands indicating demoplastic reaction.

**Figure 1d:**
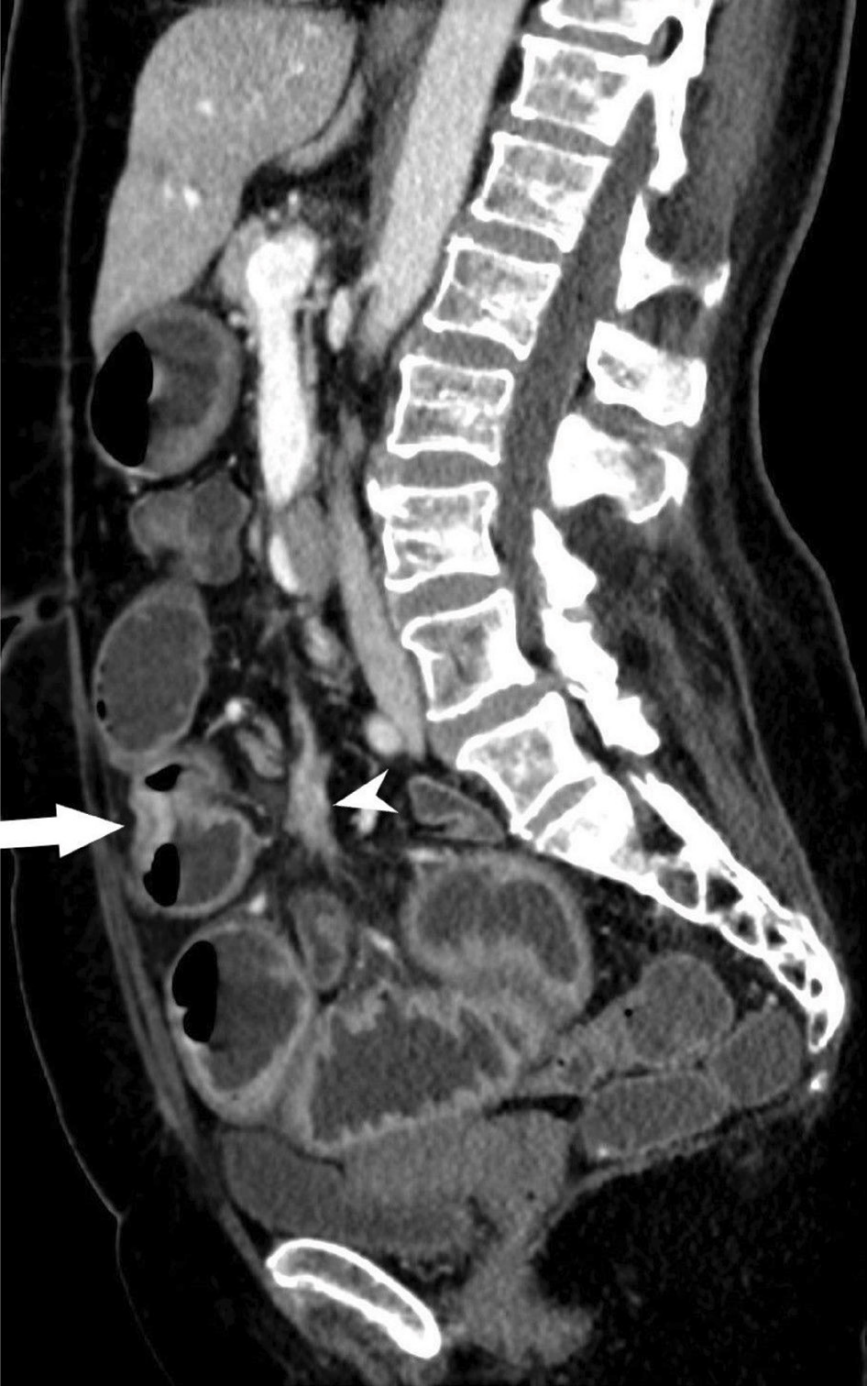
CT demonstrating stenotic ileal NET with adjacent mesenteric mass.

## Discussion

NETs of the gastrointestinal tract are uncommon endocrine tumors most commonly located in the distal small bowel. In about one-third of the cases multiple lesions are present.

The average age at diagnosis is in the fifth or sixth decade of life. Symptoms may include abdominal pain, weight loss, gastrointestinal bleeding, obstruction, ischemia, and carcinoid syndrome. Approximately 20% of patients with NETs have associated synchronous or metachronous non-carcinoid tumors, most commonly adenocarcinomas of the gastrointestinal tract.

NETs of the small bowel are submucosal, located with an intact or ulcerated overlying mucosa. A contrast-enhancing polypoid lesion protruding into the bowel lumen is the most common presentation. Less frequently, an annular lesion may be noted. Tumor infiltration and lymph node metastasis into the adjacent mesentery and local release of serotonin and other substances may result in desmoplastic reaction seen as radiating soft tissue strands resulting in angulation and retraction of bowel loops and/or encasement, thickening, and occlusion of mesenteric vessels. This may result in bowel obstruction and/or ischemia. A polypoid lesion may act as a lead point for intussusception [1].

CT enterography/enteroclysis, due to its better distension of the small bowel, has been reported to be more sensitive than conventional CT for lesion detection [1, 2]. The triad of a soft tissue density mesenteric mass, associated radiating strands, and adjacent marked contrast-enhancing bowel wall thickening has been reported to be highly suggestive of NETs. In a majority of cases, calcification may be present in the mesenteric mass.

The differential diagnosis of small bowel NETs includes adenocarcinoma, gastrointestinal stromal tumor, lymphoma, sclerosing mesenteritis, metastases, and mesenchymal neoplasm.
